# Diagnosing Lymphoma on Core Needle Biopsy and the Challenging Role of Immunohistochemistry

**DOI:** 10.7759/cureus.49983

**Published:** 2023-12-05

**Authors:** Haneen Al-Maghrabi

**Affiliations:** 1 Department of Pathology and Laboratory Medicine, King Faisal Specialist Hospital and Research Center, Jeddah, SAU

**Keywords:** large b-cell lymphoma, hematopoietic, core needle biopsy, lymphoma, immunohistochemistry

## Abstract

Publications and primary literature both describe the latest advancements in immunohistochemical diagnosis of lymphomas. Along with the updated categorizations, the growing reliance on small biopsy samples to assess lymphoma poses a constant difficulty in hematopathology diagnosis and heightens the demand for immunohistochemistry (IHC). This study aimed to provide practicing lymphoma and hematopathologists with an overview of novel immunohistochemical markers and new applications of already established immunohistochemistry markers to be helpful in lymphoma diagnosis, especially in core needle biopsies. The information was sourced from a review of relevant literature and personal experience gained through professional practice. To effectively diagnose and treat hematolymphoid neoplasms, we need to have a comprehensive understanding of the constantly evolving range of immunohistochemistry studies. This article introduced new markers that contribute to our overall knowledge of the disease, diagnosis, and treatment. The addition of these markers might be helpful in supporting the tumor diagnosis on limited sample material obtained from needle biopsies.

## Introduction and background

Zhang and Aguilera conducted a review in 2014 on the topic "New immunohistochemistry for B-cell lymphoma and Hodgkin lymphoma" [1]. They discussed the latest immunohistochemical markers for these types of lymphoma and explained how markers can be utilized for accurate diagnosis based on the 2008 World Health Organization classifications [2]. LEF1, SOX11, HGAL, LMO2, CD200, CD160, stathmin, GCET1, IRTA1, MNDA, and MYD88 were identified as new markers for small B-lymphocytic neoplasms. Additionally, c-MYC, CD200, IMP3, EB13, GCET1, and TNFAIP2 were identified as new markers specifically for large B-lymphocytic lymphomas. After this publication, numerous studies have been conducted to evaluate the efficacy of these markers and their significance in the diagnosis of lymphoma. In quick succession, two articles were published by the WHO providing updates from their 2017 revised fourth edition classification of myeloid and lymphoid neoplasms [3,4]. Additionally, another group published two articles describing an alternative international consensus classification of myeloid neoplasms, acute leukemias, and mature lymphoid neoplasms [5,6]. The diagnostic evaluation of lymphomas was enhanced in both publications by the inclusion of some of the newly discovered markers. Further analysis of English literature on B-cell lymphomas has revealed several recent research studies utilizing cutting-edge applications of known and newly developed immunohistochemistry (IHC) markers like cyclin-D3, p63, GATA3, J-chain, cortactin, MAL-RNA encoded in situ hybridization (ISH), interferon regulatory factor 8 (IRF8), and myocyte enhancer factor 2B (MEF2B). Evaluation of new T-cell markers, such as CCR4, LMO2, CD28, CD86, CD80, p-ATM, and B-cell lymphoma/leukemia 11B (BCL11B), have also been the subject of several published studies. We have discussed and assessed the possible applications and significance of these markers in our study. In addition, the utilization of IHC in diagnosing lymphoid neoplasms in tissue biopsies within a clinical setting will be discussed.

## Review

Reviewing the advancements in the utilization of new immunohistochemistry markers for B-cell lymphomas since 2014

Zhang and Aguilera previously evaluated the conventional IHC analysis for small B-cell lymphomas as mentioned earlier and presented new markers that can aid in the classification, particularly in cases with unusual IHC expression observed [1]. This can aid in the utilization of minimally invasive biopsy which pose a challenge to pathologists who are presented with limited tissue samples that may have undergone distortion due to sampling and crush artifacts. However, these newly introduced stains could potentially aid in this situation. A panel of markers, which included LEF1, MNDA, stathmin/STMN1, IRTA1, and CD27, was utilized in a tissue review of 517 small B-cell lymphomas [7]. In cases of chronic lymphocytic leukemia/small lymphocytic lymphoma (CLL/SLL), LEF1 was continually detected as positive. However, it was found to have expression in only two out of 22 marginal zone lymphoma (MZL) cases. MNDA expression was most prominent in MZL cases, although noticeable expression was observed in other types of lymphomas, such as CLL/SLL (76%), lymphoplasmacytic lymphoma (LPL) (27%), and follicular lymphoma (FL) (14%). This may restrict its usefulness in distinguishing these types of lymphomas in smaller biopsy specimens. In the assessment of FL versus splenic MZL, LPL, and other types of MZL, the level of stathmin/STMN1 expression was noteworthy. Nonetheless, in cases of CLL/SLL, stathmin/STMN1 expression (57%) was substantial and limited to paraimmunoblastic cells. IRTA1 was detected in 35% of MZL cases involving lymph nodes and in 41% of cases affecting other extranodal sites, while other types of small B-cell lymphomas exhibited minimal expression of this protein. The authors propose that the utilization of LEF1, MNDA, and IRTA1 could be beneficial in the investigation of cases with atypical IHC patterns found in diffuse patterns of small B-cell infiltrates. They also suggest implementing stathmin/STMN1, MNDA, and IRTA1 in the examination of atypical IHC patterns seen in nodular pattern small B-cell lymphomas. Another study conducted a review of the expression levels of IRTA1 and MNDA in 127 lymphomas, which included 80 cases of MZLs and 47 other small B-cell lymphomas. However, to evaluate IRTA1, they utilized it as an RNA in situ hybridization (ISH) assay [8]. Of the total MZLs examined (74), 42% (31) were found to have IRTA1 positivity in this study, with only one case of FL exhibiting the same. However, no instances of IRTA1 were detected in LPL, mantle cell lymphoma (MCL), or CLL cases. The presence of MNDA was observed in 64% (51 out of 79) of all MZLs, 37% (three out of eight) of LPLs, 21% (three out of 14) of FLs, 53% (eight out of 15) of CLLs, and 78% (seven out of nine) of MCLs. These results strengthen the recommendation for IRTA1 and MNDA usage, particularly when diagnosing MZL.

A total of 354 cases of small B-cell lymphomas were assessed for the expression of LEF1 and SOX11 [9]. The study revealed that out of 129 CLL/SLL cases, 126 (98%) exhibited positivity for LEF1. In contrast, among 33 MCL cases, only two (6%) were positive for LEF1. As for the cases of MZL and LPL that underwent examination, all 142 cases of MZL and 50 cases of LPL were found to be negative for LEF1. A recent review examined the expression of LEF1 in a group of 117 Hodgkin lymphomas, which consisted of 66 cases of classic Hodgkin lymphoma (CHL), 27 cases of nodular lymphocyte-predominant Hodgkin lymphoma (NLPHL), and 24 cases of Richter transformation of CLL/SLL to CHL [10]. LEF1 expression was detected in the majority of cases involving both Richter transformation of CLL/SLL to CHL in 80% (19 out of 24) and CHLs in 88% (58 out of 66). Therefore, we recommend not relying solely on LEF1 expression as an indicator of Richter transformation of CLL/SLL. LEF1 expression was observed in just 44% (12 out of 27) of NLPHL cases. Recently, some potential pitfalls of these previously overlooked new markers have been pointed out. MNDA is present in primary follicles, and its appearance may resemble that of MZLs, especially in cases where the biopsy is limited or the presentation is atypical. LEF1 expression is often downregulated in challenging CLL/SLL cases that are atypical or have greater prolymphocyte levels, diminishing its effectiveness as a diagnostic tool. A review was conducted on the expression of LEF1 in B-cell lymphomas other than CLL/SLL, encompassing 18 cases of MZL, LPL, and FL. Out of the two CD5-positive FLs, one was positive for LEF1, indicating potential LEF1 expression in a certain subset of FLs. However, all the (13 cases CD5 positive MZLs) and (three cases CD5 positive LPLs) were negative for LEF1 [11].

Novel immunohistochemical markers for the diagnostic assessment of B-cell lymphoma

The mounting genetic evidence implies that not only cyclin D1 but also other members of the cyclin D family (CCND2 and CCND3) could have a carcinogenic role in the pathogenesis of MCL. Around up to 15% of patients with MCL exhibit clinical, molecular, and morphologic characteristics that are comparable to those with t(11;14), despite not having the usual translocation. These cases known as MCL with cyclin D1-negative can exhibit a recognizable level of expression of either cyclin D2 or cyclin D3. Cases involving repeated translocations of CCND2 in combination with immunoglobulin heavy and light chains have been identified, which are linked to elevated levels of cyclin D2 expression. Another genetic mechanism for the overexpression of cyclin D2 or cyclin D3 involves the integration of immunoglobulin light-chain enhancers near CCND2 or CCND3, which has been observed as cryptic insertions [12]. Cyclin D3 plays a crucial role in the progression of the cell cycle from G1-phase to S-phase. It serves a critical function in the initial stages of B-cell growth and development in the bone marrow, as well as in the multiplication of B cells located in the germinal center's dark zones [13]. Multiple B-cell lymphomas have been reported to show gene mutations in the CCND3. A study examined the presence of cyclin D3 in splenic B-cell lymphoma and found that 24 out of 33 cases of splenic diffuse red pulp small B-cell lymphomas (SDRPBLs) expressing cyclin D3 [14]. In the other types of splenic B-cell lymphomas that were examined, cyclin D3 expression was infrequent. Specifically, it was not detected in any of the 40 CLLs, only in one out of seven classical Hodgkin lymphomas (HCLs) (14%), in two out of 35 MCLs (6%), and in one out of 74 SMZLs (1%).

Cortactin, a protein found in the cytoskeleton, plays role in several cellular processes such as cell motility, cellular adhesion, membrane movement, and breaking down the extracellular matrix. Carcinomas, sarcomas, and astrocytic gliomas have been reported to exhibit an increased expression of cortactin. In recent research, the expression of cortactin was examined in 131 occurrences of non-Hodgkin B-cell lymphomas and leukemias [15]. Out of the 17 CLL cases observed, positive expression was identified in 82%. However, none of the 16 MCL cases showed positive expression, whereas only 8% (two out of 25) of the FL cases and 83% (five out of six) of the MZL cases showed positivity. For extranodal MZL cases, only 41% (seven out of 17) exhibited positive expression, while only 14% (one of seven) of SMZL cases showed positivity. None of the three identified SDRPBL cases showed positivity, but all 10 of the identified HCL cases exhibited 100% positivity. Among the germinal center, diffuse large B-cell lymphoma (DLBCL) cases observed, 75% (12 out of 16) showed positivity, and among the activated B-cell-like DLBCL, 93% (13 out of 14) depicted positivity. In general, it seems that cortactin is frequently present in CLL, DLBCL, and HCL, and could serve as a useful indicator in distinguishing CLL from MCL.

While the aberrant expression of MAL in primary mediastinal large B-cell lymphoma (PMBL) was first reported in 1999, the assessment of MAL expression using available IHC staining is inadequate and not commonly utilized in pathological practice [16]. A recent study analyzed 15 cases of PMBL by utilizing MAL IHC stain and MAL RNA ISH. Results showed that 67% (10 out of 15) of PMBL cases expressed MAL according to both IHC stain and RNA ISH [17]. It is recommended to include MAL RNA ISH analysis during the evaluation of large B-cell lymphomas of the mediastinum, particularly in cases where a core needle biopsy is utilized as this is a commonly employed clinical practice. These observations imply that MAL RNA ISH can be a valuable diagnostic tool in such circumstances.

The J-chain is a protein that assists in the formation of dimers and pentads for the immunoglobulin IgA and IgM antibodies by enabling multimerization. J-chain expression is seen in reactive germinal centers and plasma cells, ranging from weak to strong staining. A study examined the use of J-chain revealing 100% strong expression in NLPHL cases (20 out of 20), while also displaying inconsistent staining in 50% of T-cell/histiocyte-rich large B-cell lymphoma (THRLBCL) (two out of four) and 67% of PMBL (10 out of 15). Interestingly, out of the 43 evaluated cases of CHL, none of them showed any positive results for J-chain. It is important to recognize that using J-chain expression alone as a diagnostic tool in core needle biopsy may create a diagnostic dilemma. Non-neoplastic germinal center B-cells and plasma cells can also express J-chain, which means that this marker cannot be relied upon solely to differentiate between the progressive transformation of the germinal center (PTGC) and NLPHL [18].

GATA3, which is associated with the maturation of T-cells, could potentially have diagnostic value in the evaluation of large B-cell lymphomas. GATA3 is expressed normally in T-cells located in paracortical and germinal center areas. When examining large B-cell lymphomas, GATA3 was found to be positively expressed in 80% of CHL, 75% in PMBL, and 100% in gray zone lymphomas (GZLs). While it shows 0% in NLPHLs, DLBCLs, and Epstein-Barr virus (EBV)-positive DLBCLs [19]. A different study analyzed the levels of p63 and GATA3 in mediastinal lymphomas to distinguish between CHL and PMBL. PMBL cases exhibited p63 expression in 94%, whereas only 15% of CHL cases showed p63 expression. In 77% of CHL cases, GATA3 expression was noted, while PMBL cases had no instances of GATA3 expression [20]. The results are in line with that of Kezlarian et al., providing more evidence for the usefulness of GATA3 in difficult mediastinal lymphoma diagnoses and revealing a potential application for p63 [19].

IRF8 functions as a nuclear transcription factor that plays a crucial role in regulating B-cell differentiation and promoting tolerance and can be detected in B-cell monocytic/monoblastic leukemia, and blastic plasmacytoid dendritic cell neoplasms. A study conducted an evaluation of IRF8 expression in CHL (74 cases), NLPHL (seven cases), ALK-negative anaplastic large cell lymphoma (ALCL) (15 cases), and ALK-positive ALCL (four cases). IRF8 was expressed in all NLPHL cases (seven out of seven), while IRF8 was expressed in 85% of CHL cases (61 out of 72). However, none of the ALCL cases showed any IRF8 expression. The results indicate that IRF8 can be utilized as a marker for B-cells to distinguish between CHL and ALK-negative ALCL, especially in very challenging cases with limited tissue material [21].

The combination of CD83, fascin, and CD23 in a panel could be beneficial for distinguishing between CHL, GZL, and PMBL in mediastinal lymphoma needle biopsies. A study examined 22 cases of CHL which displayed the presence of CD83 and fascin expression in all cases, while CD23 was detected in only two out of 22 (9%) cases. CD83 expression was found in 41% (nine out of 22) of PMBL cases, while fascin expression was detected in 32% (seven out of 22) of cases. Additionally, CD23 expression was observed in 95% (21 out of 22) of cases. GZL showed mixed expression results of CD83 in 89% (16 out of 18) of cases, fascin in 86% (18 out of 21) of cases, and CD23 in 67% (12 out of 18) of cases. The results indicate that incorporating a 3-marker panel into the diagnostic process for mediastinal lymphomas can be a valuable supplement [22].

Novel immunohistochemical markers to aid in diagnosing T-cell lymphoma

The process of assessing T-cell lymphomas through diagnosis is challenging, usually necessitating the use of a comprehensive catalog of IHC stains. Based on recent literature, it has been observed that various new markers have been evaluated to aid in this assessment, which is considered noteworthy. T-cell lineage commitment and maturation are dependent on the indispensable role played by BCL11B. In a recent investigation, the BCL11B protein expression was assessed in a set of 115 cases of T-cell acute lymphoblastic leukemia/lymphoblastic lymphoma (T-ALL/LBL) that encompassed various subtypes, such as early T-cell precursor acute lymphoblastic leukemia/lymphoma (ETP-ALL/LBL) (29 cases) (25%), early T-ALL/LBL (26 cases) (23%), thymic T-ALL/LBL (42 cases) (37%), and mature T-ALL/LBL (18 cases) (16%). Results showed that the majority of ETP-ALL cases (83%) did not show BCL11B expression, while the majority of non-ETP-ALL/LBL cases (84%) showed positive BCL11B expression. Moreover, patients who were diagnosed with positive BCL11B in ETP-ALL demonstrated a favorable survival rate compared to those who had negative BCL11B expression (p=0.009). In general, determining the level of BCL11B expression could be useful in distinguishing ETP-ALL/LBL cases from other types of T-ALL/LBL. Additionally, BCL11B has the potential to be utilized as a prognostic indicator for patients with ETP-ALL [23].

LMO2 is a protein that plays a role in supporting transcription factors (such as the GATA family) required for hematopoiesis and angiogenesis. Identifying T-lymphoblastic lymphoma (T-LBL) can be challenging, especially when it occurs in conjunction with myeloid/lymphoid neoplasms with eosinophilia, as it is an aggressive form of T-lymphoid precursors that is rarely seen [24]. LMO2 is highly expressed in the majority of T-LBLs, whereas it is not observed in undeveloped TdT-positive T cells in the thymus or slow-growing T-lymphoblastic growths. Out of 11 cases of T-LBLs linked to myeloid/lymphoid neoplasms with eosinophilia, a study found that nine of them did not have LMO2, indicating a distinct molecular process for T-LBLs associated with myeloid/lymphoid neoplasms with eosinophilia [25]. Interestingly, LMO2 expression is observed in lymphomas derived from germinal center lymphocytes, including FL, Burkitt lymphoma (BL), and DLBCL, as well as in NLPHL [18].

For other T-cell lymphomas, which are composed of heterogeneous groups with evolving classifications, the usage of IHC in core needle biopsy might not be helpful in reaching a definitive final diagnosis. Thus, we recommend pathologists use a scheme algorithm based on the result of CD30. We recommend using a set of T-cell panels such as CD2, CD3, CD5, and CD7. The next set of IHCs are as follows: CD30, CD4, CD8, and EBV-ISH. T-cell lymphomas can be categorized into two main groups, based on the degree of expression of CD30. The neoplastic cells in the first group show strong and consistent expression of CD30; these can be designated as CD30-positive T-cell lymphoma. Those in the second group who exhibit either a lack of expression or partial expression of CD30 can be categorized as CD30-negative T-cell lymphoma. This can be very useful to patients with T-cell lymphomas expressing CD30 which will show high response rates to the anti-CD30 antibody conjugate brentuximab [26]. When the CD30 marker shows a strong positive presence in nearly all lymphoma cells, the subsequent course of action involves assessing the level of ALK (CD246) expression. The presence of a positive ALK result confirms the diagnosis of ALK-positive ALCL. Yet, caution should be taken in the small-cell variant ALCL, which departs from the usual CD30 expression pattern found in the other variants, where most lymphoma cells are small and do not express CD30. However, only a small subset of large lymphoma cells with hallmark cell morphology exhibits a positive CD30 expression. A variety of neoplasms could be considered as potential diagnoses if ALK is detected as negative. These could include ALK-negative ALCL, mycosis fungoides (MF) with CD30 positive expression/large-cell transformation, adult T-cell leukemia/lymphoma, primary cutaneous ALCL, and breast implant-associated ALCL. Additionally, a subgroup of peripheral T-cell lymphoma-not otherwise specified (PTCL-NOS) that exhibits significant CD30 expression in over 80% of lymphoma cells could also be considered as a potential viable diagnosis.

**Figure 1:**
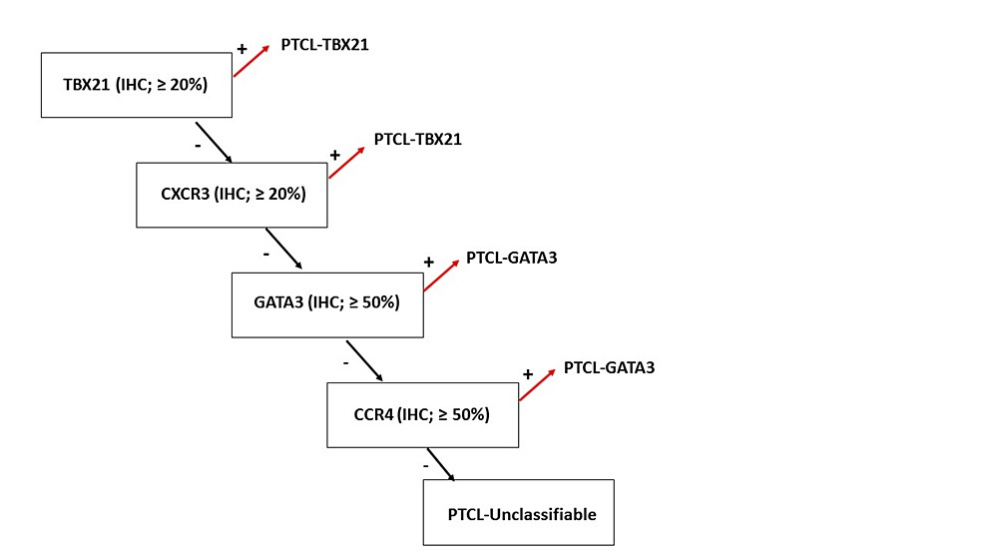
The proposed algorithm by Amador et al. to utilize immunohistochemistry to identify the cell of origin in PTCL-NOS. In T-cells, the differentiation pathways of Th1 and Th2 are primarily regulated at a transcriptional level by TBX21 and GATA3, which act as master regulators. TBX21 and GATA3 regulate the expression of CXCR3 and CCR4, which are associated with Th1 and Th2 phenotypes as cytokines. In the absence of the four specific immunomarkers, the instance is classified as unclassifiable PTCL. In the absence of the four specific immunomarkers, the instance is classified as unclassifiable PTCL. The positivity threshold for TBX21 and CXCR3 was set at 20%, whereas for GATA3 and CCR4 it was 50%. PTCL-NOS: peripheral T-cell lymphoma-not otherwise specified; IHC: immunohistochemistry The image is adapted with permission from Amador et al. [28].

New insights into PTCL-NOS have been gained through gene expression profiling studies. Two significant molecular subgroups of PTCL-NOS were discovered, wherein one demonstrated overexpression of TBX21 (TBET) (known as PTCL-TBX21), while the other exhibited overexpression of GATA3 (known as PTCL-GATA3). Each subgroup exhibits unique biological variations in how they use oncogenic pathways and how this impacts their prognosis [27]. As suggested by Amador et al., the initial step of the proposed immunohistochemical algorithm involves identifying TBX21 expression, which may be followed by evaluating CXCR3, GATA3, and CXCR4 if necessary (Figure 1) [28]. Patients belonging to the PTCL-GATA3 subgroup and a specific group within the PTCL-TBX21 subtype that has a cytotoxic nature are associated with an exceptionally unfavorable prognosis, with a survival rate of less than a year. However, patients diagnosed with PTCL-TBX21 have a median survival rate that surpasses two years [27].

## Conclusions

To diagnose and treat lymphoid neoplasms, it is essential for pathologists and hematopathologists to possess knowledge of the constantly expanding range of immunohistochemistry techniques. The diagnosis can be difficult at times due to the limited material obtained through needle core biopsy. However, this article presents new markers that can aid in our comprehension of the disease, diagnosis, and treatment.
